# Right subclavian artery to right atrium bypass using Polytetrafluoroethylene (PTFE) graft in hemodialysis patient with central venous occlusion: Case report

**DOI:** 10.1016/j.amsu.2022.103438

**Published:** 2022-03-03

**Authors:** Abdellah Rezziki, Hicham El malki, Sara Boukabous, Youssef Banana, Hicham Meftah, Intissar Haddiya, Yassamine Bentata, El Mehdi Moutaouekkil, Adnane Benzirar, Omar El Mahi

**Affiliations:** a Department of Vascular Surgery, Mohammed VI University Hospital of Oujda, Mohammed First University of Oujda, Morocco; b Department of Cardiovascular Surgery, Mohammed VI University Hospital of Oujda, Mohammed First University of Oujda, Morocco; c Department of Nephrology – Dialysis and Kidney Transplantation, Mohammed VI University Hospital of Oujda, Mohammed First University of Oujda, Morocco; d Laboratory of Epidemiology, Clinical Research and Public Health, Faculty of Medicine and Pharmacy, Mohammed the First University of Oujda, Morocco

**Keywords:** Hemodialysis, Central venous occlusion, Vascular access, Bypass, Atrium, Subclavian artery

## Abstract

**Introduction:**

Central venous Occlusion (CVO) is a serious complication that occurs mainly in patients with long term central venous catheters for dialysis. It remains a challenge in vascular surgery.

**Case presentation:**

We report a case of a patient with end-stage kidney disease (ESKD), who was admitted for chronic occlusion of the superior and inferior vena cava and underwent a right subclavian artery to right atrium (RA) bypass using polytetrafuloroetylene (PTFE) graft.

**Clinical discussion:**

Central venous catheters remains the main cause of CVO in ESKD. Although the endovascular therapy is the main approach in the treatment of CVO, the surgical bypass to the RA is often the last resort to preserve vascular access in hemodialysis patients. The autologous vein and bovine arterial bypass remains better than PTFE grafts in terms of long term patency.

**Conclusion:**

fistulas as a first approach for dialysis access must be privileged at the expense of central catheters. However bypass to RA by mini thoracotomy incision remains as an excellent option for dialysis access in ESKD with CVO.

## Highlights

• Central venous Occlusion (CVO) is a serious complication which occurs mainly in patients with chronic indwelling central venous catheters for dialysis.
• We report the case of a patient with end-stage Kidney disease (ESKD), who was admitted for chronic occlusion of the superior and inferior vena cava. So she underwent a right subclavian artery to right atrium (RA) bypass using polytetrafuloroetylene (PTFE) graft.
• Bypass to RA by mini thoracotomy insision is rarely performed, and remains the last ditch resort to preserve adequate vascular access in hemodialysis patients with central venous occlusion.
• Fistulas as a first approach for dialysis access must be privileged at the expense of central catheters to prevent CVO.

## 1 Introduction

Maintaining the vascular access in patients with end-stage KIDNEY disease (ESKD) is a challenge in vascular surgery. Central venous Occlusion (CVO) of the superior and inferior vena cava is a serious complication that occurs mainly in patients with chronic indwelling central venous catheters for hemodialysis or for pharmaceutical interventions [1].

Endovascular treatment including angioplasty and stenting is certainly the main treatment, but its failure leads to surgical practice [1].

Anterior chest wall grafts for hemodialysis access were first described in the 1970s [2], and are performed in patients who have consumed their primary and secondary approaches.

We report a case of right subclavian artery to right atrium subcutaneous chest wall graft created using a mini thoracotomy incision in a patient with significant arterial disease alongside with superior and inferior venous cava occlusion.

Our case report was written according to SCARE guidelines [3].

## 2 Case Presentation

We report a case of a 54 -year –old female with ESKD undergoing hemodialysis since 2015, who has thrombosed all the native approaches in the upper limb, reason why, she underwent an arteriovenous bypass which subsequently thrombosed with failure of thrombectomy. Thus we have made a native arterio-venous fistula (AVF) of the right lower limb which subsequently thrombosed without the possibility of thrombectomy. Given the difficulty of maintaining her vascular accesses, the patient benefited on several occasions from central venous catheters.

At admission, the patient benefited from extremities venograms which showed chronic occlusion of the superior and inferior vena cava and major tributaries.

The staff's decision was to perform a loop bypass between the right axillary artery and the right atrium with the cardiac surgery team.

After approaching the right axillary artery at the level of the delto-pectoral sulcus, we discovered a poor quality artery with little pulse.

So we converted to an approach of the right subclavian artery, which was in good quality. A right anterior minithoracotomy was performed at the level of the third intercostal space with ligation of the artery and the internal thoracic vein. Exploration objectified a pericardium slightly stuck to the heart. After pericardiotomy and exposure of the right atrium by fixation wires, lateral clamping of the right atrium and subclavian artery were performed.

**Fig. 1:**
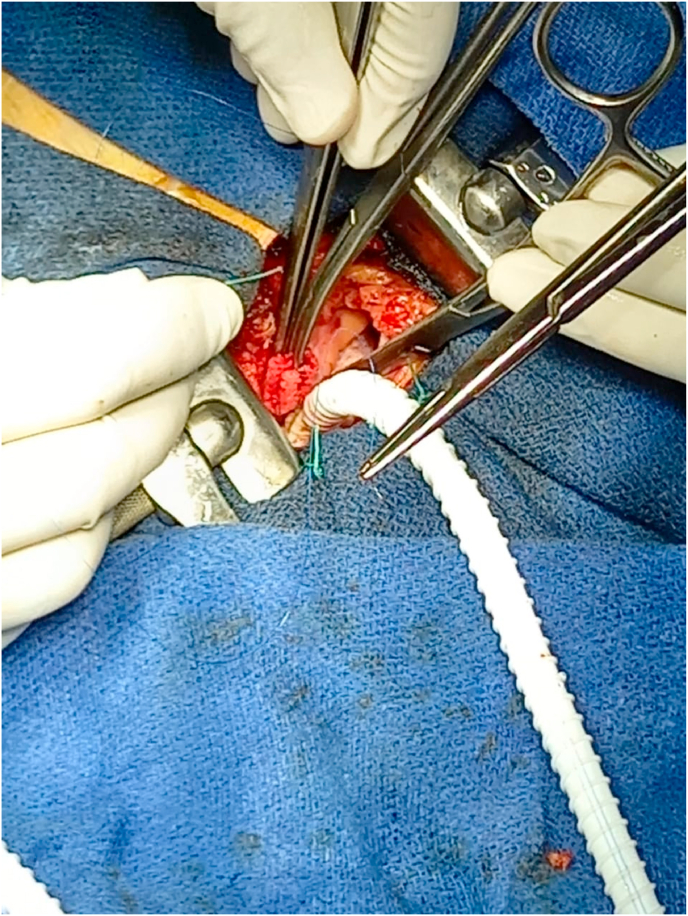
First anastomosis between the right atrium and the armed prosthesis with 5/0 prolene.

**Fig. 2:**
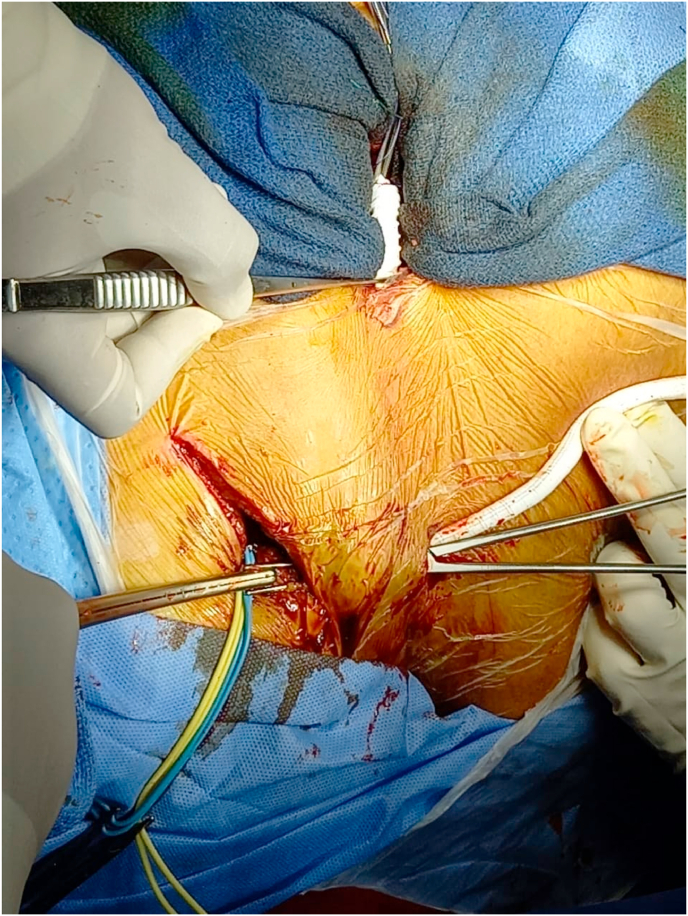
Tunneling of the prosthesis facing the third intercostal space jostling towards the right subclavian artery.

After systemic heparinization (50/UI kg), we realized a first anastomosis between the right atrium and the armed prosthesis with 5/0 prolene (Fig. 1). After tunneling of the prosthesis facing the third intercostal space jostling towards the right subclavian artery (Fig. 2), a 2 cm longitudinal arteriotomy was made and a spatulated end-to-side anastomosis was completed with running 5-0 monofilament polypropylene suture (Prolene).

After unclamping, we have noticed good flow, with a strongly palpable thrill over the graft.

The surgical management of this case was performed by an experienced professor of vascular surgery with the aid of an attending and 2 junior residents.

Immediately after surgey, the patient was transferred to the intensive care unit, and put on curative anticoagulation (low molecular weight heparin 0,6 UI/12h), and on antiplatelet aggregation.

The patient was pricked through her bypass, 2 weeks after surgery, without difficulty.

A treatment based on antiplatelet aggregation was prescribed on discharge of the patient; with regular follow up at our specialized consultation.

## 3 Discussion

ESKD patients who have Consumed all their peripheral access, and who presents CVO remains a challenge for all vascular surgeons.They most often have central venous catheters, which make them vulnerable to CVO. Central venous catheters leads to endothelial cell aggression, with the formation of a thrombus and consequently a CVO [4]. That's why Kidney Disease Outcome Quality Initiative (KDOQI), recommends catheter last and fistula first for dialysis access.

Endovascular therapy is the first line approach to the treatment of central venous occlusion and SVC syndrome, but its failure leads to surgical practice [5].

The surgical bypass to the RA is often a last ditch resort to preserve adequate vascular access, for patients with inadequate peripheral veins.

The department of vascular surgery at Houstton methodist hospital, reported good outcomes concerning bypass to the RA, using bovine carotid artery conduit [6] and autologous femoropopliteal vein [7].

After we had discussed this case with our colleagues of cardiac surgery department, we decided to perform a central venous bypass using polytetrafluoroethylene (PTFE) graft, since the patient doesn't have an autologous vein suitable for bypass surgery,which long term patency is better than PTFE grafts [8].

In turn, bovine arteriel bypass is considered as superior to PTFe one, as it is described in the literature, that biological grafts seems to perform better than PTFe grafts in patients with thrombophilia [6].

Nevertheless, multiple cases of axillary artery to RA bypass using PTFe graft have been reported [9,10], besides it remains the only way of rescue in patients with CVO, who don't have a suitable autologous vein for bypass, furthermore we did not have a bovine arteriel available at our institute.

This central venous bypass was performed using a mini thoracotomy, which remains an excellent substitute for the median sternotomy.

According to Morsy and al, anterior chest wall grafts have primary and secondary patency, as well as other forms of autogenous or complex access [11]; moreover, since the skin is thick in this area of body, the risk of infection is low, besides the canulation is easy. [12].

Owing to the developed collateral circulation around the subclavian artery, ischemic complications after axillary atrial bypass are exceptional.

## 4 Conclusion

Fistulas as a first approach for dialysis access must be privileged, besides central catheters should not be considered as a hemodialysis access except in emergency situations,thereby we can decrease CVO. However bypass to RA by mini thoracotomy incision remains an excellent option for dialysis access in ESKD with CVO.

## Sources of funding

There's no financial support

## Ethical approval

Applicable.

## Consent

Obtained.

## Author contribution

A. Rezziki: CONCEPTION, LITERATURE REVIEW, ANALYSIS, DATA COLLECTION, WRITING- REVIEW & EDITING.  H. El Malki: CONCEPTION, LITERATURE REVIEW, ANALYSIS, DATA COLLECTION, WRITING- REVIEW & EDITING. S.Boukabous: CONCEPTION, LITERATURE REVIEW, ANALYSIS, DATA COLLECTION, WRITING- REVIEW & EDITING. Y. Banana: CONCEPTION, LITERATURE REVIEW, ANALYSIS, DATA COLLECTION, WRITING- REVIEW & EDITING. H.Meftah: CONCEPTION, LITERATURE REVIEW, ANALYSIS, DATA COLLECTION, WRITING- REVIEW & EDITING. I.Haddiya: CONCEPTION, METHODOLOGY, SUPERVISION.  Y.Bentata: CONCEPTION, METHODOLOGY, SUPERVISION.  El M. Moutaouekkil: CONCEPTION, METHODOLOGY, SUPERVISION. A. Benzirar: CONCEPTION, METHODOLOGY, SUPERVISION.  O. El Mahi: CONCEPTION, METHODOLOGY, SUPERVISION.

## Research registration

This is not an original research project involving human participantsin an interventional or an observational study but a case report. This registration is not required.

## Guarontor

Dr. Youssef Banana (corresponding author): 00212658641363/yousef.bana18@gmail.com (Department of vascular surgery, Mohammed VI University Hospital of Oujda, Mohammed premier University of Oujda, Morocco)

## Provenance and peer review

Not commissioned, externally peer-reviewed.

## Declaration of competing interest

There is no conflicts of interest between the authors.
